# Residual susceptibility to measles among young adults in Victoria, Australia following a national targeted measles-mumps-rubella vaccination campaign

**DOI:** 10.1186/1471-2458-7-99

**Published:** 2007-06-08

**Authors:** Heath A Kelly, Heather F Gidding, Theo Karapanagiotidis, Jennie A Leydon, Michaela A Riddell

**Affiliations:** 1 Epidemiology Unit, Victorian Infectious Diseases Reference Laboratory (VIDRL) and World Health Organization (WHO) Western Pacific Region Measles Reference Laboratory, Melbourne, Australia; 2 School of Population Health, University of Melbourne, Australia; 3 Centre for Infectious Diseases and Microbiology (CIDM)-Public Health, Westmead Hospital, Australia; 4 Serology Laboratory, VIDRL and WHO Western Pacific Region Measles Reference Laboratory, Melbourne, Australia

## Abstract

**Background:**

Past measles immunisation policies in Australia have resulted in a cohort of young adults who have been inadequately vaccinated, but who also have low levels of naturally acquired immunity because immunisation programs have decreased the circulation of wild virus. A measles-mumps-rubella (MMR) immunisation campaign aimed at addressing this susceptibility to measles among young adults was conducted in Australia in 2001–2. By estimating age-specific immunity, we aimed to evaluate the success of this campaign in the state of Victoria.

**Methods:**

We conducted serosurveys after the young adult MMR program at state and national levels to estimate immunity among young adults born between 1968–82. We compared results of the Victorian (state) surveys with the Victorian component of the national surveys and compared both surveys with surveys conducted before the campaign. We also reviewed all laboratory confirmed measles cases in Victoria between 2000–4.

**Results:**

The Victorian state serosurveys indicated no significant change in immunity of the cohort following the young adult MMR campaign (83.9% immune pre and 85.5% immune post campaign) while the Victorian component of the national serosurvey indicated a significant decline in immunity (91.0% to 84.2%; p = 0.006). Both surveys indicated about 15% susceptibility to measles among young Victorian adults after the campaign. Measles outbreaks in Victoria between 2000–4 confirmed the susceptibility of young adults. Outbreaks involved a median of 2.5 cases with a median age of 24.5 years.

**Conclusion:**

In Victoria, the young adult MMR program appears to have had no effect on residual susceptibility to measles among the 1968–82 birth cohort. Young adults in Victoria, as in other countries where past immunisation policies have left a residual susceptible cohort, represent a potential problem for the maintenance of measles elimination.

## Background

Reflecting previous disease circulation and vaccination policies, it was recognised that, by the end of the twentieth century in Australia, measles had become a disease mostly affecting young adults [1,2]. The first major measles outbreak in Australia involving predominantly young adults occurred in Victoria between February and May 1999. Approximately 85% of the 75 notified cases confirmed with measles in this outbreak were born between 1968 and 1981 (aged between 18 and 31 years at the time) [2]. These young adults were most likely to be susceptible to infection because of the timing of changes to measles vaccination practices in Australia. Measles vaccine was first licensed in Australia in 1968, recommended for children aged 15 months in 1971, and included for 12-month-old infants in the first national childhood immunisation schedule in 1975. Measles-mumps vaccine was introduced in Victoria in February 1983 and measles-mumps-rubella (MMR) was introduced in 1989 [3]. Prior to the introduction of vaccine, most people acquired immunity through infection with wild measles virus in childhood. Despite suggestions of initially poor uptake [4], the availability of measles vaccine in Australia from 1968 lead to a reduction in circulating wild measles virus, reflected in decreased measles and measles encephalitis admissions to Victoria's former infectious diseases hospital [5].

As a consequence of changes to vaccination policies, a proportion of the 1968–82 birth cohort had not been exposed to either wild or vaccine virus. Since 1999 measles outbreaks in Victoria have involved predominantly young adults and unimmunised children. In three large measles outbreaks in Victoria between 1999 and 2002, the median age of cases has varied between 22 and 25 years [2,6,7]. Estimates of susceptibility to measles in the 1968–82 birth cohort ranged from 15–21% from two surveys with sera collected in 1999, one using blood donors and the other using residual diagnostic sera stored at the Victorian Infectious Diseases Reference Laboratory [1]. Estimated measles susceptibility at a national level was 9% [8]. In recognition of residual measles susceptibility among young adults, the Commonwealth Department of Health funded an MMR immunisation program in the financial year 2000–2001. The program targeted adults aged 18–30 years [9]. Funding was provided to the states for the purchase of vaccine for young adults who visited their general practitioner in 2001–2. There were no specific funds for planning or advertising the campaign and there was no provision, at the time, for any campaign evaluation. Some states ran programs that targeted tertiary students [10] but young working adults were not directly targeted.

This approach was very much in contrast to the national measles control campaign, which had been conducted in 1998 targeting school-aged children. The campaign was coordinated by the Commonwealth Department of Health and had appropriate planning and resources to provide school-based vaccinations. It resulted in an estimated 94% measles immunity in children aged 6–11 years and 91% in children aged 12–18 years[11].

In order to provide an outcome evaluation of the young adults measles campaign, the Commonwealth Department of Health funded a national serosurvey in 2002, performed by the National Centre for Immunisation Research and Surveillance. An independent state-based serosurvey was performed in Victoria. Our aim was to estimate measles immunity in young Victorian adults following the measles immunisation program and to compare the results from the national and state serosurveys. Post campaign serosurvey immunity estimates were compared with previously published pre-campaign estimates [1,8]. We also reviewed all laboratory confirmed cases of measles in Victoria between 2000 and 2004.

## Methods

### Samples and sample size

Young adults targeted by the MMR campaign were aged approximately 17–31 years in 1999, at the time of the first large outbreak, and in the range 20–34 years in 2002. The population aged 20–34 years in Victoria in 2002 was 1,056,474. We estimated measles immunity of Victorian residents aged 20–34 years in 2002 (born 1968–82) one year after the young adult immunisation program. For the state-based evaluation, we used residual diagnostic sera collected in 2002 and stored at the Victorian Infectious Diseases Reference Laboratory. We compared the results for the 2002 sample with previously published results for 1999 [1]. For the national evaluation, we compared the sera submitted by Victorian diagnostic laboratories to two national serosurveys in 1996–9 [8] and 2002 [12].

The estimated sample size for the 1999 state-based serosurvey was 246, based on 80% immunity with 5% precision for a 95% confidence interval [1]. The 2002 state-based serosurvey assumed age-specific immunity would be unchanged and the sample size estimate was the same. The sample size for the national serosurvey in 2002 was calculated to detect a 5% difference in immunity in young adults from the first serosurvey with 80% power and 95% confidence [12]. National sample sizes were not intended to be able to detect differences between states.

### Assay protocol

Testing for the state-based surveys was performed at the Victorian Infectious Diseases Reference Laboratory and for the national surveys at the Institute for Clinical Pathology and Medical Research in Sydney. Testing was done using the Enzygnost (Dade Behring, Marburg, Germany) commercial measles IgG enzyme immunoassay (EIA) in both laboratories. Using the alpha formula provided, the manufacturer's instructions for the calculation of immunoglobulin class G (IgG) concentration based on optical density (OD), were followed and immunity was determined as OD >= 0.2, equivalent to approximately 340 milli-International Units per millilitre (mIU/ml). Equivocal samples were retested and reclassified as positive or negative, where indicated. Final equivocal results were counted as non-immune.

In the state-based serosurveys, serum samples that had been submitted for measles or rubella testing were excluded from all study samples and all serum samples were anonymised. Ethics approval for the anonymous testing of residual diagnostic sera has been obtained from the Royal Melbourne Hospital Research Foundation Human Ethics Committee. In the 2002 national serosurvey, sera excluded were those from patients known to be immunocompromised, HIV positive, or those with a history of multiple transfusions in the three months prior to sample collection. In the 1996–9 national serosurvey, samples that had been tested for measles were also excluded. Appropriate state and local ethics committees approved the national serosurveys [11,12].

### Analysis

Given the study design, there was no information available about the vaccination history of individuals whose sera were tested. We assumed measles vaccination policies had affected measles immunity in the population and further assumed that population age-specific immunity would be reflected in the convenience samples. Analysis of measles immunity was therefore based on the presumed effect of changes in measles immunisation policy on the 1968–82 birth cohort. Samples from the state-based study were analysed in two birth cohorts. For both state-based serosurveys we analysed birth cohorts that reflected changes made to immunisation policy affecting infants aged 12 months in 1968–73 and 1974–82. Victorian samples from the national serosurvey were analysed as one birth cohort, 1968–82.

The state-based sample included equal numbers of each age and the proportion positive by age group was calculated as all positive divided by all tested. Because a larger number of sera were tested from younger ages in both national samples, age group specific estimates of immunity from the national sample were directly adjusted to the age distribution of the Australian population in 2002. Results for both serosurveys were imported into STATA version 8 for analysis (StataCorp, College Station, Texas, USA). Exact 95% confidence intervals for proportions were calculated and Fisher's exact test was used to assess the difference in proportions.

### Laboratory confirmed measles in Victoria

An attempt is made to confirm all measles notifications in Victoria by laboratory diagnosis at the Victorian Infectious Diseases Reference Laboratory, the state, national and WHO regional reference laboratory for measles. We reviewed all laboratory confirmed measles cases on the laboratory database between 2000 and 2004 and distinguished between clusters and sporadic cases, based on epidemiological information gathered as part of enhanced surveillance in Victoria [13] and genotyping of cases [14,15].

## Results

### Immunity following the young adults MMR campaign, state-based serosurveys

Of all samples, 5.4% and 3.6% were equivocal in the 1999 and 2002 state-based serosurveys respectively and were classified as not protected. Following the young adults MMR campaign, there was a small non-significant increase in overall immunity from 83.9% (95% CI, 79.4–87.8) to 85.5% (95% CI, 80.8–89.4) between 1999 and 2002, but no evidence of any significant increase in the proportion immune in either of the 1968–73 or the 1974–82 birth cohorts (Table 1). However the older birth cohort (1968–73) was more likely to be immune in both serosurveys, with differences in the proportion immune of 8.6% (95% CI 0.7–16.5) and 9.3% (95% CI 1.5–17.1) for the 1999 and 2002 serosurveys respectively.

**Table 1:** State-based estimates of measles immunity amongst young adults in Victoria before (1999) and after (2002) the young adult measles immunisation program

Measles vaccine	Birth cohort	Age 1999 (years)	N	Percent immune (*95% CI*)	Age 2002 (years)	N	Percent immune (*95% CI*)	p-value 1999 vs 2002
Licensed 1968	1968–1973	26–31	120	89.2 *(82.2–94.1)*	29–34	112	91.1 *(84.2–95.6)*	0.79

Scheduled at age 12 months in 1975, until 1983 when measles-mumps vaccine was introduced	1974–1982	17–25	191	80.6 *(74.3–86.0)*	20–28	170	81.8 *(75.1–87.3)*	0.67

Total	1968–1982	17–31	311	83.9 *(79.4–87.8)*	20–34	282	85.5 *(80.8–89.4)*	0.65

### Immunity following the young adults MMR campaign, national serosurveys

Ten Victorian laboratories contributed a range of 40–68 serum samples per laboratory to the 1996–9 serosurvey, compared with seven laboratories contributing a range of 16–111 samples per laboratory to the 2002 serosurvey. The Victorian Infectious Diseases Reference Laboratory contributed a greater proportion of sera in the 2002 serosurvey (34% in 2002 compared with 12% in 1999; Table 2). In each serosurvey, estimates of immunity from sera stored at the Victorian Infectious Diseases Reference Laboratory were lower than all except one other laboratory, although no differences were statistically significant. Excluding the Victorian Infectious Diseases Reference Laboratory, four Victorian laboratories contributed reasonable sample numbers in both surveys; three had lower point estimates of immunity in the 2002 serosurvey and one had a higher point estimate. Confining estimates of immunity between surveys to these four laboratories and the Victorian Infectious Diseases Reference Laboratory resulted in an estimated decrease in immunity in the 1968–82 birth cohort from 90.4% to 81.9% (p < 0.001), similar to the estimated decrease when all available data were included (Table 2).

**Table 2:** National serosurvey estimates of measles immunity amongst young adults (born 1968–1982) in Victoria before (1996–9) and after (2002) the young adult measles immunisation program by laboratory

	1996–9 serosurvey*				2002 serosurvey			
Laboratory	Samples tested	Percent of all samples tested (%)	Percent measles protected by laboratory^† ^(%)	Samples tested	Percent of all samples tested (%)	Percent measles protected by laboratory^† ^(%)	Change in percent protected (%)	p-value for change
VIDRL**	45	12.2	88.1	111	34.0	80.5	-7.65	0.2
Lab 2	68	18.4	90.4	46	14.1	80.6	-9.78	0.2
Lab 3	55	14.9	90.1	66	20.2	88.5	-1.62	0.6
Lab 4	40	10.8	86.7	16	4.9	96.9	10.16	0.3
Lab 5	43	11.6	95.7	38	11.7	83.3	-12.41	0.2
Other labs	118	32.0	91.2	5	1.5	80.0	-11.2	0.5

Total	369	100	91.0	282	100	84.2	-6.80	0.006

* 1996–8 serosurvey used for the 1968–80 birth cohort; 1999 serosurvey used for the 1981–2 birth cohort

^† ^age-adjusted to the 2002 Australian population

** Victorian Infectious Diseases Reference Laboratory

### Laboratory confirmed measles in Victoria

After the large outbreak involving 75 cases in Victoria in 1999 [2], there were 16 outbreaks involving more than one case in the state between 2000 and 2004. The maximum number of cases in any one outbreak was 22, while 20 cases were not associated with any further transmission. The median number of cases per outbreak, including sporadic cases, was 1 and, for outbreaks involving more than one case, was 2.5. The median age of all laboratory confirmed cases in this period was 24.5 years.

## Discussion

Estimates from the national and the state-based serosurveys of the proportion of young adults born between 1968–82, resident in Victoria and immune to measles in 2002, were in close agreement (84.2% and 85.5%, respectively). Neither serosurvey provided evidence of increased immunity in young adults following the targeted MMR campaign. The important difference in the serosurveys was the higher estimate of young adult immunity in Victoria from the first national serosurvey. An apparent decline in immunity among young Victorian adults in the national serosurveys, evident in all except one of the Victorian laboratories but not evident from the state-based serosurveys, is not easy to explain on a population basis.

In the results of the national evaluation, other states did not demonstrate a decrease in immunity among young adults between serosurveys and immunity in Victoria in 1996–9 was similar to other states [12]. The higher estimate of young adult immunity in the first national serosurvey may be a sampling anomaly. We have no other explanation for the finding. However we have previously shown no significant population health difference in susceptibility to a number of vaccine preventable diseases when comparing the 1996–9 national sample of residual diagnostic sera from Victoria with sera obtained from a three-stage random cluster survey in Victoria, suggesting that residual diagnostic sera are representative of the population [16].

A decline in immunity in young Victorian adults, if it were a valid finding, might suggest waning vaccine induced immunity. Based on a small pilot study of 25 young adults tested over a median period of approximately six years, we found no evidence of waning immunity between surveys. However we have no information on the vaccination or exposure history of the individuals tested and cannot draw any significant conclusions from this observation. Given the low levels of circulating measles virus in Victoria following interruption of endemic measles virus transmission [14,15], it would be expected that changes in antibody levels would reflect vaccination history. In a cohort study from Luxembourg of waning measles and rubella immunity with follow-up over 6.8 years, it was suggested that, in the absence of booster vaccination, measles antibody decay following vaccination was about 2.4% per year [17]. Several other studies have demonstrated a decline in antibody titre over time following vaccination but this has not been reported to occur in naturally infected individuals [18-20].

Whether there has been a decline in immunity or not, both studies conclude that residual susceptibility to measles among young Victorian adults remains around 15% after the young adult MMR immunisation program. The measles notification rate in Victoria has fluctuated since the 2002 serosurvey and, although the rate was low in 2004 (0.3 per 100,000), the median age of notified cases was 26 years [21]. The ongoing problem of residual susceptibility to measles among the young adult population, first evident in Victoria from a number of outbreaks and confirmed by serosurveys, has subsequently been demonstrated by outbreaks affecting mainly young adults in other Australian states [22-25].

The failure of the young adult measles campaign can be attributed to the lack of central coordination; a very limited plan for targeting this age group through general practice visits; no budget for promoting the campaign; and a fragmentation of strategies to engage and vaccinate the target group in various states. In addition MMR doses distributed to the target population were not monitored and vaccine coverage in the target population could not be measured. A centrally coordinated and well-planned young adult vaccination campaign, targeting both males and females aged 15–39 years with a measles-rubella (MR) vaccine, was successful in Costa Rica in May 2001. MR coverage achieved in the campaign was 87% in the 30–34 year age group and greater than 90% in all other target age groups [26]. The target population was 1,606,329, about 60% larger than the target population in Victoria.

## Conclusion

For reasons similar to those in Australia, young adults remain susceptible to measles in other developed countries, such as the United States [27] and France [28]. While it may be possible to maintain measles elimination in these countries in the short term, residual susceptibility in identified cohorts will need to be addressed to maintain measles elimination and global eradication in the longer-term [29].

## Competing interests

The author(s) declare that they have no competing interests.

## Authors' contributions

HK conceived the study, performed the analysis of the Victorian data and drafted the manuscript. HFG assisted in refining the study concept, performed the analysis of the Victorian data from the national serosurveys and contributed to drafting the manuscript. TK performed all serological assays for the Victorian data. JAL supervised the serological assays in Victoria and was responsible for the contribution of sera from the Victorian Infectious Diseases Reference Laboratory to the national serosurveys. MR contributed to study design, data analysis and preparation of the manuscript. All authors read and approved the final version of the manuscript.

## Pre-publication history

The pre-publication history for this paper can be accessed here:


